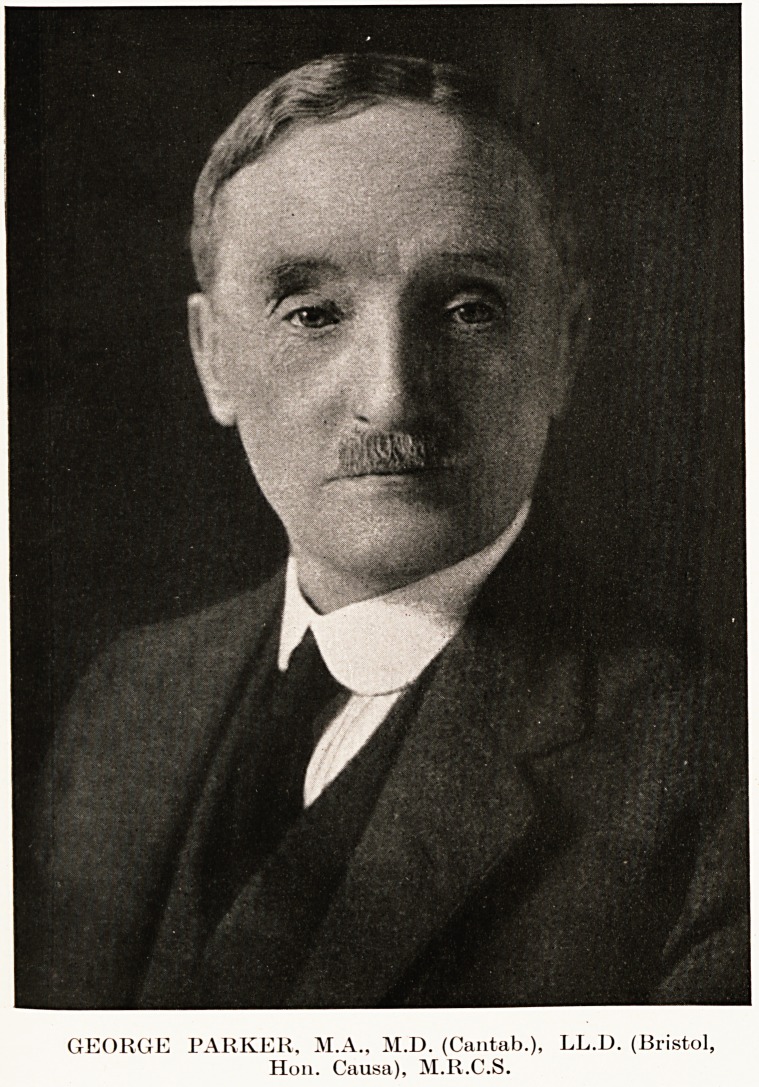# George Parker

**Published:** 1937

**Authors:** 


### Obituary

GEORGE PARKER, M.A., M.D. (Cantab.), LL.D. (Bristol Hon. Causa), M.R.C.S.

ON 27th April George Parker died at his house in Pembroke Road, aged 84 : one of the most distinguished of the Alumni of our University and Medical School, full of years, beloved and honoured by all who knew him or with whom at any time he had been associated.

He was the son of George Parker, of Claverdon Hall, West Bromwich, and born in the year 1853. He passed from School

GEORGE PARKER, M.A., M.D. (Cantab.), LL.D. (Bristol, Hon. Causa), M.R.C.S.

at Stratford-on-Avon to St. John's College, Cambridge ; he obtained a Second Class in the Moral Science Tripos, 1876, and the next year Second Class in the Historical Tripos. He was an Assistant Master at Rugby for a short time and then went to St. Bartholomew's Hospital and, having qualified as M.D., M.R.C.S., he studied for a while at Vienna.

He began practice in Bristol when in 1887 he was appointed a Medical Officer at the Bristol Dispensary. It was then the writer began almost a lifelong friendship with this endearing man. He was appointed Assistant Physician at the Bristol General Hospital, and later full Physician, and in due course he was elected Honorary Consulting Physician.

George Parker was also Lecturer and Examiner in Medical Jurisprudence at the University of Bristol, as well as Consulting Physician to the Bristol Dispensary, and member of the Association of Physicians, and later was President of the Bath and Bristol Branch of the British Medical Association.

His rather small house in Pembroke Road at those times, where he lived unmarried and devoted to his rather delicate, much-loved sister, was stored with so many over-filled book-shelves that gave his study at times an air of gloom, till he entered and made one feel that the contact, whether short or long, seem of all too fleeting minutes.

He gave his best skill and attention to poor and rich alike, but fortunately had time enough for the literary and historical researches that have laid the medical world under an obligation which we acknowledge so thankfully. The bibliography which is appended proves his industry as a skilful observer and consulting physician. Yet his outstanding genius was perhaps shown most in his many historical papers and contributions, the outcome of long years of reading and research.

He was attached to the Second Southern Hospital, with the rank of Lieut.-Col., R.A.M.C.(T.), where for the duration of the Great War he rendered great service.

When on 2nd July, 1934, George Parker was accorded the great honour of an Honorary LL.D. by the University of Bristol, assembled in the Great Hall, the public orator, Professor Crofts, admirably crystallized his more outstanding literary achievements, and " Eminently worthy of the Degree of Doctor of Laws."

" I present to you in Dr. George Parker one who has devoted the energies of more than forty-five years to the service of medicine in this city, and was for many years Lecturer in Medical Jurisprudence in our University.

" He is eminent not only as a practising physician, and late

President of the Bath and Bristol Branch of the British Medical Association, but also as a historian of medical and surgical practice.

"His *Survey of Early Surgery in Great Britain*, and his *History of the Barber Surgeons* are well known to students, and he was appointed by the Royal College of Surgeons to deliver the Vicary Lecture in 1927 on the subject of 'Early Hospitals.' His *History of the Bristol Medical School* was published last year, when the school celebrated its centenary.

"In this happy combination of medical skill and humane scholarship Dr. Parker fitly embodies one of the traditional characters of a profession which has produced many men eminent in literature and in the arts. I present him to you as an old friend of this University; a man distinguished in his calling."

One must add to these honours that he was an Honorary Member of the Bristol Medico-Chirurgical Society of Bristol, of which he had been President in 1915–16, and President of the Association of Physicians of Great Britain and Ireland in 1924, at the Bristol Meeting. His Presidential Address, "Medical Organization and Growth of the Medical Sciences in the Seventeenth Century," illustrated by the lives of local worthies, is a fascinating compilation, making a delightfully-told narrative that can never be out of date, and naturally of peculiar interest to his fellow-Bristolians. What adds so much to the historical value of all Parker's contributions is his constant reference to his sources of information, hence in telling his story he gives a sense of truth and actuality that is too often lacking in such contributions.

A glance at the lengthy enumeration of his chief writings and contributions reveals not only his industry in recording valuable observations throughout many years, but also his exceptional versatility. One may be excused if one directs particular attention especially to some of his historical contributions, because in this sphere he was unsurpassed, the "History of Barber Surgeons in Great Britain," International Congress of Medicine, 1913, "Early History of Surgery in Great Britain," "Medical Organization of Science in the Seventeenth Century," and the allegory of "Robinson Crusoe."

One must, however, refer to other channels of his scientific activities, such as the Cambridge Antiquarian Society and the Bristol and Gloucestershire Archæological Society.

As Honorary Medical Librarian of the Medico-Chirurgical Library at the University of Bristol he rendered most valuable services, so unobtrusively given throughout many years. As an archæologist his " Supplementum Chirurgiæ " appeared in the *Proceedings of the Clifton Antiquarian Club*, 1902, and his interesting paper, " A Visit to the Near East," 1927, related to his work with Professor Flinders Petrie, and he was an enthusiastic member of the Bristol and Gloucestershire Archæological Society and likewise of the Cambridge Antiquarian Society.

We would mention one of what may be termed the minor contributions, viz. : *The History of the Hospital of St. John Baptist, Redcliffe Pit, Bristol*, because it serves to show how much is possible with a wonderful gift of imagination in one with a zest for digging out facts on which to erect a solid structure, for " the story of this once famous and beloved house is not easy to trace." Parker shows how our hospital can be traced to the time of Henry II, and its wealth seems to have come down to the Grammar School and to the Redcliffe School. As with all of George Parker's contributions, this arrests attention and commands interest. It is a good example of his extraordinary constructive work when what appears only worthless old relics became in his hands the key to re-light historical caves.

It was only very near the close of his long years of wonderful activity that George Parker's energies appeared to wane, and at the end he was tended with devotion by the Clifton Down Convent Nursing Sisters, whom he had served for many years as a devoted physician and ever a warm-hearted, very human friend.

One feels that too little has been told of George Parker himself, but it is impossible to portray our colleague without recounting his life's interests, and of the especially valuable historical work which probably no one else could do, at least so well.

A man without an enemy, one ever ready to help a friend, and to aid and cheer any patient. Quiet, self-composed, ever working and pursuing.

Canon S. F. Alford, Chairman of the Committee of the Clifton Dispensary, writes : " I gladly bear my testimony to Dr. George Parker. He was linked up with us as a member of the Committee for many years, and since 1918 had acted as Honorary Consulting Physician of the Institute. Although a busy man, he was one of the most regular members at our meetings, and was seldom absent. Within a few weeks of his death he was with us as usual. He took a keen interest in the work of the Dispensary, and his advice was valued. He was always ready to respond to the call of duty and faithful in giving his best to any service in which he was engaged, and won the affection of all his poor patients. He will be greatly missed."

A tribute from Sister Angelina, Mother Superior of the Clifton Down Convent (Nursing Sisters), Litfield Place, Clifton: "Doctor George Parker had the gift of penetrating into his patients' feelings, echoing them in his sympathetic heart with great tenderness and compassion, and adding to the physician's abilities the magic power of goodness. Moreover, he was endowed with a most honest mind, inspiring in anyone approaching him the strength of noble feelings. During the thirty years he attended to our dear invalid priests and community, I constantly admired his great understanding, particularly toward those afflicted ministers and his untiring philanthropy to mankind. It was with great delight that in return for such kindness and at his own request we sent our sisters to comfort his declining years. This, whilst giving us the opportunity to bestow on him our tender care, was in itself a precious token of his life-long appreciation of our devoted nursing sisters. His name is included in our list of benefactors. I hope I have made myself clear about our everlasting gratitude to Dr. Parker. Though I am French and my English not too good, I have tried to convey my personal opinion of a great physician."

From C. H. Walker, M.B., F.R.C.S.:—

It was in the summer of 1890 that I first met George Parker. He invited me, then a stranger in Bristol, to dine with him at his house in Pembroke Road, and from that day onward he became my constant friend. I found he knew a relative of mine who had been at the same college with him but was two years his junior. Parker tried, unsuccessfully, to persuade this freshman to join the Cambridge University Rifle Volunteers, nicknamed the "Bug Shooters." It is not recorded that Parker was ever much of a marksman, but that he should have been a volunteer at all will be a surprise to those who only knew him in later life. He had already taken honours in two triposes when he began his medical studies, so his name appeared in the Cambridge pass examinations at the head of the list with a letter M before it. In consequence he was known by other students as "Magister Parker." He never engaged in any games or sport (though I have known him play family whist), but he loved the country, and I often accompanied him on long country walks. His wide and varied knowledge made him a most interesting companion. He seemed to know all about the owners of property, the diseases of cattle and wayside botany. When he was lecturer on Materia Medica he used to take immense pains to get fresh specimens of the various plants and ranged the country far and wide. Since many plants were not in season at the time of his lecture, he made a collection of dried specimens, and was a good deal chaffed about his "hortus siccus."

I hardly know if Parker had any hobbies, unless they were the acquisition of knowledge and doing acts of kindness. For six years I lived next door to him in Pembroke Road. When my children were small he always invited them to his Christmas party, which they enjoyed immensely. He used to come to us to tea on Christmas Day, and always brought a present mysteriously secreted under his overcoat, which my children came to look forward to as a matter of course.

His patients also trespassed on his kindness, for he was prepared to call and see them at all sorts of times, no matter how busy or how fatigued he was. Sometimes in his long walks into the country he would call on a patient (often a hospital one) whom he had befriended. In later years he still took these long walks, usually alone, and one afternoon, when he was close upon eighty years of age, I met him on Failand Hill trudging wearily to Clevedon to see a friend. I was in my car, and knew that on his way over Cadbury Camp he would pass my house, so I begged him to let me give him a lift. He steadfastly refused, but consented to come to tea. About an hour later he turned up, looking still more weary. After only a short rest he plodded on again, and completed the eleven mile walk to Clevedon safely.

George Parker had no enemies. Everyone was his friend, and many loved him dearly.

---

MEDICAL PAPERS AND ADDRESSES BY GEORGE PARKER.

"A Sarcoma in the Common Fowl," *Brit. M. J.*, 1883, ii. 431.
"On Vertigo: a Critical Digest," *Brain*, 1885, vii. 514.
"Early Tracheotomy in Diphtheria," *Brit. M. J.*, 1887, i. 1274.
"A Case of Hystero Epileptoid Attacks," *Brain*, 1887, ix. 546.
"Two Cases of Bronchial Fistula," *Bristol Med.-Chir. J.*, 1893, xi. 89.

"Reports on Contemporary Medicine," *Bristol Med.-Chir. J.*, 1894 to 1910, and in 1913 to 1920.

"The Use of Calcium Chloride," *Clin. J.*, 1894–1895, v. 344.

"Localized Œdema," *Hospital*, 1895, 397.

"On the Use of Coley's Fluid in Sarcoma," *Bristol Med.-Chir. J.*, 1895, xiii. 313.

"Hæmato-Porphysinuria and Plumbism," *Bristol Med.-Chir. J.*; 1897, xv. 213.

"Extreme Bradycardia" (Stokes Adams d.) with D. G. Fendick, *Bristol Med.-Chir J.*, 1899, xvii. 113.

"A Case of Hydrochloric Acid Poisoning," 1899.

"Malignant Diseases of Œsophagus," *Brit. M. J.*, 1898, i. 399.

"The Diagnosis of so-called Rheumatic Diseases," *Lancet*, 1897, i. 1735.

"The Early Races who Inhabited Britain," an Address given at the Literary and Philosophical Club, Bristol.

"Micro-organisms in Disease," an Address at the Scientific Club.

"Cannibalism," an Address at the Scientific Club, 189.

Abstracts of Current Medical Literature for *The Hospital* for ten years, in conjunction with P. Watson-Williams and others.

"Chronic Joint Diseases as seen in Medical Out-patients," *Hospital*, 1899, xxv. 397.

"The Various Forms of Meningitis," *Hospital*, 1900, xxviii. 167.

"Congenital Hepatic Cirrhosis and Obliteration of Bile-ducts," *Lancet*, 1901, ii. 520.

"Cystic Kidney," *Am. J. M. Sc.*, 1899, cxviii. 272.

"Splenic Leukæmia and Phthisis in the same patient," *Brit. M. J.*, 1902, i. 1,136.

"Mongolian Imbeciles," *Bristol Med.-Chir. J.*, 1900, xviii. 81.

"Aneurism of Transverse Aorta," *Bristol Med.-Chir. J.*, 1900, xviii. 78.

"Recent Applications of Electricity," *Hospital*, 1902, xxxiii. 93.

"The Supplementum Chirurgiæ," *Proc. Clifton Antiq. Club*, 1902.

"General Pheumococcal Infection," *Brit. M. J.*, 1903, i. 1,081.

"Bronzed Diabetes," *Brit. M. J.*, 1903, ii. 1,052.

"Notes on Cases in an Electrical Department," *Bristol Med.-Chir. J.*, 1904, xxii. 112.

"Some Disputed Points in Pleurisy," an Address before the Dispensary Clinical Society.

"Infantile Paralysis: a Clinical Lecture," *Hospital*, 1906, xl. 311.

"Causes of Paralysis of the Third Nerve," *Hospital*, 1907, xli. 123.

"The Estimation of Pulse Tension," *Hospital*, 1907, xli. 439.

"Splenic Leukæmia and Gout in the same patient," *Brit. M. J.*, 1907, i. 1,170.

"Causes of Transient Cerebral Paralysis," *Bristol Med.-Chir. J.*, 1909, xxvii. 15.

"The Belfast Meeting of British Medical Association," *Bristol Med.-Chir. J.*, 1909, xxvii. 269.

"Notes on Biliary and Intestinal Sand," *Bristol Med.-Chir. J.*, 1910, xxviii. 112.

"Uræmia with Intestinal Ulcers," *Lancet*, 1910, ii. 1,134.

"Epidemic of Infantile Paralysis (37 cases) in Bristol," *Brit. M. J.*, 1911, i. 609.

"Medical Organization and Progress of Medical Sciences in the Seventeenth Century," Presidential Address to Bath and Bristol Branch of B.M.A., *Bristol Med.-Chir. J.*, 1911, xxix. 201.

"National Insurance Act," *Brit. M. J. Supplement*, 1912, ii. 262, 558.

"Recent Researches on Poliomyelitis," *Hospital*, 1913, liii. 587.

"The Barber Surgeons," *Bristol Med.-Chir. J.*, 1912, xxx. 327.

"History of the Barber Surgeons in Great Britain," International Congress of Medicine, London, 1913.

"Doctors and State Insurance," *Daily Chronicle*, 1912, Feb. 13th.

"A Case of Veronal Poisoning" (with Mr. E. Russell), *Brit. M. J.*, 1914, i. 853.

"A Case of Woody Phlegmon of the Neck," *Brit. M. J.*, 1914, i. 24.

"The Founding of the Medical-Chirurgical Society, and the History of Military Medicine in England," Presidential Address, *Bristol Med.-Chir. J.*, 1915, xxxiii. 129.

"Some Aspects of High Blood Pressure," *Clin. J.*, 1916, xlv. 357.

"The Province of Medical Ethics," *Edinburgh M. J.*, 1918, xxi. 191.

"Recurrent Fever due to Bacillus Fæcalis," *Lancet*, 1917, ii. 837.

"Milk Substitutes (in War time, Liquid Oatmeal)," *Brit. B. J.*, 1918, i. 53, 736.

"The Diagnosis of Paralysis of the Upper Extremity," a Post-Graduate Lecture, *Clin. J.*, 1920, xlix. 1.

"A Case of Erythræmia or Splenic Polycythæmia," *Bristol Med.-Chir. J.*, 1920, xxxvii. 91.

"The Prevalent Diseases of the World," *Nursing Times*, 1920, April.

*The Early History of Surgery in Great Britain*, published in *Medical History Manuals*, A. & C. Black Ltd., 1920.

"Mental Effects of Encephalitis Lethargica," *J. Neurol. & Psychopath.*, 1920, i. 223.

Editor (with Dr. J. F. Brickdale) of Michell Clarke's Neurological and other Papers, published by J. W. Arrowsmith Ltd., 1920.

"Early Bristol Medical Institutions and Ordinances of Bristol Surgeons," *Trans. Bristol and Gloucestershire Archæol. Soc.*, 1922, xliv. 155.

"Osteoarthritis of the Hip-Joint," *Brit. M. J.*, 1922, ii. 539.

"The History of the Bristol Medical School," *Nonesuch*, 1923, ix. 37.

*Notes on Forensic Medicine*, Part I, published by Taylor Bros., Bristol, 1925.

*The Hospital of St. John Baptist, Bristol: A Study of Existing Records*, published by St. Stephen's Press, 1925.

"Uveo-Parotitic Paralysis," *Bristol Med.-Chir. J.*, 1926, xliii. 73.

Lists of Medical Men in Bristol from the earliest times to 1925 (unpublished).

"The Divorce of Surgery from Medicine," *Brit. M. J.*, 1925, ii. 582.

An article on "Bristol" in *The Book of Bath*, 1925.

"Notes on a Visit to the Near East," *Bristol Med.-Chir. J.*, 1927, xliv. 173.

"The Early Development of Hospitals. The Vicary Lecture before the Royal College of Surgeons, 3rd Nov., 1927. Published in *Brit. J. Surg.*, 1928, xvi. 39.

"Summary of Church Councils Checking Practice of Law and Medicine by Ecclesiastics," *Cath. Med. Guardian*, 1928, April.

"Discovery of Anæsthetic Powers of Nitrous Oxide," *Lancet*, 1928, i. 60.

Lecture to Bristol Painters: "Lead and Other Poisons," 1928, Feb. 9th.

"Progressive Muscular Atrophy, Peroneal Type (21 relatives affected)," *Brit. M. J.*, 1928, i. 1,062.

"English Medicine: Its History and Progress," *Canad. J. Med. & Surg.*, 1930, lxviii. 44.

"The History of Tyndall's Park, Fort Royal and House," *Trans. Bristol and Gloucestershire Archæol. Soc.*, 1929, li. 123.

### ADDRESSES AND PAPERS ON VARIOUS SUBJECTS.

"The Allegory of Robinson Crusoe," *History*, 1925, x. 11.

"British Agriculture, Comparisons and Figures," *Bristol Times and Mirror*, 1917, 5th May.

"Development of English Church History, 1525–1900," Lecture to the C.E.M.S., 1918.

"The Road-mender's Thoughts," *Bristol Times and Mirror*, 1925, June 24th.

"The Services of the Eastern Orthodox Church."

"Yell Fara as a Mirror of History."

"The Evidence for the Resurrection."

"'Happy Thought' Cot Protector," *Lancet*, 1899, i. 459.

"Traffic on the Severn in the Eighteenth Century."

"The Griffin," *Reminiscences*, Lower School of L.S., Rugby.

## Figures and Tables

**Figure f1:**